# Effect of Neoadjuvant Chemotherapy in Patients with Resectable Colorectal Liver Metastases

**DOI:** 10.1371/journal.pone.0086543

**Published:** 2014-01-21

**Authors:** Dexiang Zhu, Yunshi Zhong, Ye Wei, Lechi Ye, Qi Lin, Li Ren, Qinghai Ye, Tianshu Liu, Jianmin Xu, Xinyu Qin

**Affiliations:** 1 Department of General Surgery, Zhongshan Hospital, Fudan University, Shanghai, China; 2 Department of Liver Surgery, Zhongshan Hospital, Fudan University, Shanghai, China; 3 Department of Oncology, Zhongshan Hospital, Fudan University, Shanghai, China; Indiana University School of Medicine, United States of America

## Abstract

**Background:**

Whether patients with resectable colorectal liver metastases (CRLM) receive survival benefit from neoadjuvant chemotherapy remains controversial.

**Methods:**

We retrospectively analyzed 466 patients with resectable CRLM between 2000 and 2010. Patient characteristics and survival data were recorded.

**Results:**

The patients were divided into one group with neoadjuvant chemotherapy (group NC, n = 121) and another without (group WN, n = 345). There was no difference in 5-year survival (52% vs. 48%) between the two groups. No significant differences were identified between the two groups in terms of 30-day mortality (1.7% vs. 1.2%) or morbidity (33.9% vs. 25.8%). A primary tumor at stage T4, ≥4 liver metastases, the largest liver metastasis ≥5 cm in diameter, and a serum CEA level ≥5 ng/ml were independent prognostic factors. By assigning one point to each, the patients were divided into a low-risk group (0–2) and a high-risk (3–4). The patients in the low-risk group received no survival benefit from neoadjuvant chemotherapy, whereas those in the high-risk group received survival benefit (5-year survival, 39% vs. 33%, P = 0.028).

**Conclusions:**

Preoperative neoadjuvant chemotherapy did not increase mortality or complications. Not all resectable patients, only those with >2 independent risk factors, received survival benefit from neoadjuvant chemotherapy.

## Introduction

Surgical resection of liver metastases offers the only chance for curing patients with colorectal liver metastases (CRLM), although the proportion of surgery is only 10–15% [1]. Recently, multidisciplinary discussions and advancements in chemotherapy, targeted therapy, surgery, and locoregional treatment have increased resectability and profoundly improved survival [2]. Approximately two-thirds of patients will suffer recurrence after liver surgery, so there has been increasing interest in the use of perioperative systemic chemotherapy [3]. However, whether preoperative neoadjuvant chemotherapy is appropriate for resectable CRLM remains controversial. Therefore, we retrospectively assessed the combination of preoperative neoadjuvant chemotherapy, surgery and postoperative adjuvant chemotherapy, relative to the combination of surgery and postoperative adjuvant chemotherapy in the patients with resectable CRLM who were admitted to Zhongshan Hospital between 2000 and 2010.

## Patients and Methods

Between January 2000 and December 2010, 466 initially resectable patients treated with curative surgery for CRLM were identified at Zhongshan Hospital. Based on their exposure to neoadjuvant chemotherapy, those patients were divided into a group with neoadjuvant chemotherapy (group NC, n = 121) and a group without neoadjuvant chemotherapy (group WN, n = 345). Patient and tumor characteristics as well as surgical procedure and survival data were extracted from the CRLM Database of Zhongshan Hospital. All patients of the CRLM Database provided written informed consent. This retrospective study was approved by the Institutional Review Board of Fudan University School of Medicine.

Standard demographic and clinicopathologic data were collected from each resectable patient. Details regarding mortality and complications were obtained from the CRLM Database and, when necessary, from patient records. The date of the last follow-up visit and vital signs were also collected from the CRLM Database. The median follow-up time was 34.5 months.

Categorical data were compared using the chi-square test, and continuous data with the independent-samples t-test. Survival rates were calculated using the life-table method, and compared with Kaplan-Meier survival curves and log-rank tests. Factors that were associated with survival in the univariate analyses (with an inclusion criterion of P<0.05) were entered into a multivariate analysis to test for significant effects. The specific contribution of prognostic variables was examined by means of a multivariate Cox's proportional hazards model. P<0.05 was considered significant. All statistical calculations were performed using SPSS software, version 16.0 (SPSS Inc., Chicago, IL, USA).

## Results

### Clinicopathologic Characteristics

Of 466 initially resectable CRLM patients, 121 received preoperative neoadjuvant chemotherapy, and 345 did not. Those resectable patients received preoperative chemotherapy or not based on the multi-disciplinary team (MDT) discussion. The clinical and pathologic characteristics of the two groups are outlined in Table 1. There were no significant difference in gender, age, interval from primary to liver metastases, location, differentiation, T stage, N stage of primary cancer, preoperative CEA level, number of involved liver lobes, number, size of liver metastases or concomitant with extrahepatic metastases between two groups. In addition, 121 resectable patients got 1–6 cycles (median, 4) of neoadjuvant chemotherapy, and the chemotherapy regimen included FOLFOX (n = 76), XELOX (n = 17), FOLFIRI (n = 21) and others (n = 7). There was no patient who became unresectable due to progression during neoadjuvant chemotherapy. There were 115 patients (115/121) who had adjuvant chemotherapy in group NC, and 332 (332/345) in the group WN. And the two groups were comparable in postoperative chemotherapy.

**Table 1:** Clinicopathologic characteristics of patients with resectable CRLM.

	NC Group n = 121	WN Group n = 345	P
Male: female	81∶40	213∶132	>0.05
Median age (years)	58.0 (35–72)	59.0 (28–84)	>0.05
Interval from primary to liver metastases			>0.05
≤6 months	61(50.4%)	202(58.6%)	
>6 months	60(49.6%)	143(41.4%)	
Primary tumor			>0.05
Rectum	40(33.1%)	134(38.8%)	
Colon	81(66.9%)	211(61.2%)	
Differentiation			>0.05
I–II	62(51.2%)	178(51.6%)	
III–IV	59(48.8%)	167(48.4%)	
T stage			>0.05
T1–3	45(37.2%)	141(40.9%)	
T4	76(62.8%)	204(59.1%)	
N stage			>0.05
N0	39(32.2%)	118(34.2%)	
N1–2	82(67.8%)	227(65.8%)	
CEA (ng/ml) median (range)	20.8 (4.9–779.6)	14.5 (0.1–1000.0)	>0.05
Number of involved liver lobes			>0.05
Unilobar	91(75.2%)	284(82.3%)	
Bilobar	30(24.8%)	61(17.7%)	
Number of liver metastases			>0.05
1–3	72(59.5%)	235(68.1%)	
≥4	49(40.5%)	110(31.9%)	
Diameter of largest liver metastasis (cm) median (range)	6.0 (2.5–20.0)	3.5 (1.0–18.0)	>0.05
Number of largest liver metastasis ≥5 cm	57(47.1%)	146(42.3%)	>0.05
Concomitant with extrahepatic metastases	19(15.7%)	36(10.4%)	>0.05

### Incidence of Mortality and Morbidity

The postoperative 30-day mortality was 1.7% (2/121) in the NC group, and 1.2% (4/345) in the WN group, with no significant difference between the groups. The incidence of morbidity in the NC group was 33.9% (41/121): 20.7% (25/121) had hepatic complications, and 13.2% (16/121) had systemic complications. In the WN group, the prevalence of morbidity was 25.8% (89/345): 13.6% (47/345) had hepatic complications, and 12.2% (42/345) had systemic complications. There was no significant difference in morbidity between the two groups, although a trend toward higher morbidity was observed in the NC group (Table 2).

**Table 2:** Mortality and morbidity of resectable patients.

	NC Group n = 121	WN Group n = 345	P
Mortality	2 (1.7%)	4 (1.2%)	>0.05
Total morbidity	41 (33.9%)	89 (25.8%)	>0.05
Hepatic complications	25 (20.7%)	47 (13.6%)	
Hemorrhage/hematoma	2	2	
Bile leakage	4	8	
Transient hepatic insufficiency	3	4	
Ascites	11	22	
Subphrenic fluid	3	7	
Other	2	4	
Systemic complications	16 (13.2%)	42 (12.2%)	
Pleural effusion	6	21	
Pneumonia/atelectasis	4	8	
Urinary tract infection	1	4	
Other	5	9	

### Overall Survival

**Figure 1:**
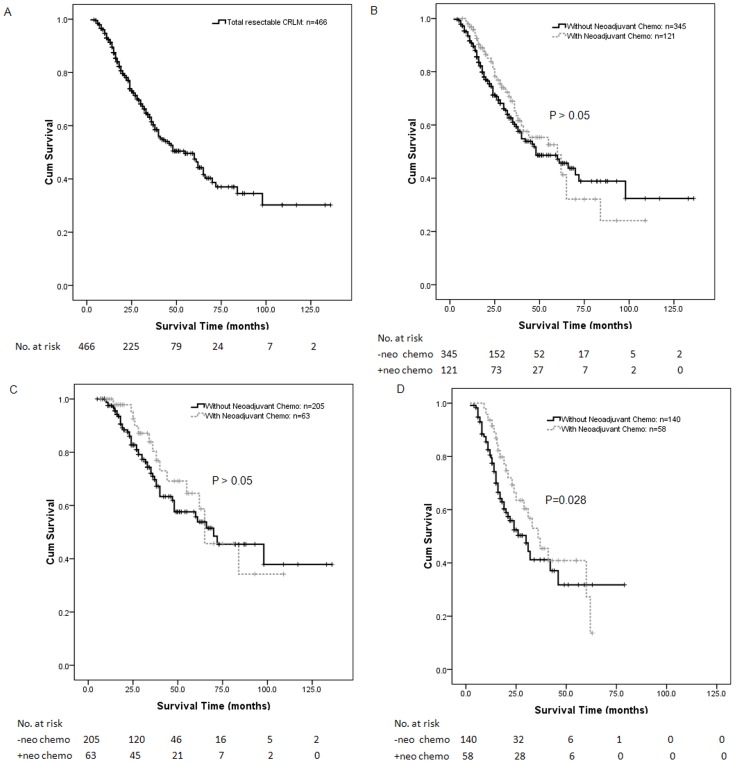
Survival of patients with resectable CRLM after liver resection. Fig. 1A Kaplan–Meier curves illustrating survival of all patients with resectable CRLM after liver resection. A total of 466 resectable patients had a median survival of 56.1 months and a 5-year survival rate of 49%. Fig. 1B Kaplan–Meier curves illustrating survival of resectable patients with or without neoadjuvant chemotherapy. 121 patients with neoadjuvant chemotherapy had a median survival of 60.0 months and a 5-year survival rate of 52%, whereas those without neoadjuvant chemotherapy had a similar median survival of 53.4 months and a 5-year survival rate of 48%. Fig. 1C Kaplan–Meier curves illustrating survival of low-risk patients with or without neoadjuvant chemotherapy. In the low-risk group, the patients with and without neoadjuvant chemotherapy had similar survival (median survival, 60.0 m vs. 60.0 m; 5-year survival, 64% vs. 57%; P>0.05). Fig. 1D Kaplan–Meier curves illustrating survival of high-risk patients with or without neoadjuvant chemotherapy. In the high-risk group, patients who had received neoadjuvant chemotherapy had a better overall median survival (38.9 m vs. 28.4 m) and a more favorable 5-year overall survival (39% vs. 33%; P = 0.028) than those had not received neoadjuvant chemotherapy.

The 466 patients had a median survival of 56.1 months, and the 5-year survival rate was 49% (Fig. 1A). Among the 121 patients in the NC group, the median overall survival was 60.0 months, and the 1-, 3-, and 5-year overall survival rates were 97%, 69%, and 52%, respectively. In comparison, the patients in the WN group had a similar median overall survival (53.4 months; P>0.05) and 5-year overall survival rate (48%; P>0.05) (Fig. 1B).

### Univariate and Multivariate Analyses

In the univariate analyses, several clinicopathologic factors were found to be associated with survival (Table 3). Female sex, a primary tumor at stage T4, an N-positive primary tumor, ≥4 liver metastases, the largest liver metastasis ≥5 cm in diameter, and a serum CEA level ≥5 ng/ml were associated with increased risk of death. In the multivariate analysis, the 6 factors listed above were analyzed using a multivariate Cox proportional hazard regression model. A primary tumor at stage T4, ≥4 liver metastases, the largest liver metastasis ≥5 cm in diameter, and a serum CEA level ≥5 ng/ml were found to be independent predictors of poor survival (Table 3).

**Table 3:** Clinicopathological characteristic associated with overall survival.

Variable	n	Median Survival (mo)	5-yr Survival Rate	Univariate	Multivariate
Gender				0.033	0.107
Female	172	45.0	46%		
Male	294	60.0	50%		
Age (years)				0.302	/
<70	386	60.0	50%		
≥70	80	35.7	39%		
Disease-free interval from primary to liver metastases				0.220	/
≤6 months	263	47.5	46%		
>6 months	203	60.0	52%		
Differentiation of primary tumor				0.061	/
I–II	240	60.0	50%		
III–IV	226	51.3	47%		
Invasion of primary tumor				0.012	0.019
T1–3	186	60.0	58%		
T4	280	42.0	42%		
Regional lymph nodes of primary tumor				0.036	0.610
N0	157	60.0	57%		
N1–2	309	45.3	43%		
Number of involved liver lobes				0.555	/
Unilobar	375	56.1	49%		
Bilobar	91	55.6	49%		
Number of liver metastases				0.012	0.009
<4	307	60.0	57%		
≥4	159	37.5	35%		
Diameter of largest liver metastasis				<0.001	<0.001
<5 cm	263	60.0	62%		
≥5 cm	203	33.9	30%		
Serum CEA at diagnosis of liver metastases				<0.001	0.012
<5 ng/ml	118	60.0	62%		
≥5 ng/ml	348	44.3	44%		
Extrahepatic metastases				0.292	/
No	411	60.0	51%		
Yes	55	39.4	33%		
Preoperative neoadjuvant chemotherapy				0.088	/
No	345	53.4	48%		
Yes	121	60.0	52%		

By assigning one point to each of the above 4 independent risk factors, all of the resectable patients were divided into a low-risk group (0–2) and a high-risk group (3–4). There were 268 patients in the low-risk group and 198 in the high-risk group. In the low-risk group, patients who had received neoadjuvant chemotherapy and those who had not received neoadjuvant chemotherapy had similar survival (median survival, 60.0 m vs. 60.0 m; 5-year survival, 64% vs. 57%; P>0.05) (Fig. 1C). In the high-risk group, the patients who had not received neoadjuvant chemotherapy had a median survival of 28.4 m and a 5-year survival rate of 33%, whereas those who had received neoadjuvant chemotherapy had a better overall median survival (38.9 m) and a more favorable 5-year overall survival rate (39%; P = 0.028) (Fig. 1D).

## Discussion

Over half of colorectal cancer patients ultimately develop liver metastases, and surgical resection presently offers the best opportunity for a cure and a positive outcome [4]. In the past decade, the proportion of CRLM patients viewed as amenable to resection has increased as surgeons have become more aggressive and systemic therapy has become more effective [5]. These patients can receive survival benefit from liver surgery, with an overall 5-year survival of 40–55% [6]–[8]. Consistent with the literature, the resectable patients had a 5-year survival rate of 49% after liver surgery in the current study.

In patients with resectable CRLM, both postoperative adjuvant and perioperative chemotherapy appear to be beneficial over surgery alone [9], [10]. The EORTC Intergroup trial 40983 by Nordlinger and colleagues demonstrated that perioperative chemotherapy with FOLFOX4 is compatible with major liver surgery, and reduces the risk of relapse by a quarter, with more reversible postoperative complications compared to surgery alone [11]. However, a subsequent analysis suggested that this did not translate into an improved overall survival benefit, but it must be noted that this study was never powered to test for such an improvement in overall survival, which remains a secondary end point of the study [12]. Aiming to identify baseline factors that may predict a survival benefit, a further exploratory retrospective analysis showes that perioperative FOLFOX seems to particularly benefit patients with resectable CRLM when CEA is elevated and performance status is unaffected, regardless of the number of metastatic lesions [13]. However, whether those patients receive a survival benefit from preoperative neoadjuvant chemotherapy remains unknown.

Neoadjuvant chemotherapy prior to hepatectomy in patients with resectable CRLM may increase the resectability of liver lesions, treat occult metastasis, improve progression-free survival, allow for testing of the chemosensitivity of the cancer in situ, help to determine the appropriateness of further treatments, and identify progressive disease that contraindicates immediate surgery [14]. An Italian study of 25 patients with primarily resectable CRLM treated with neoadjuvant chemotherapy found that 18 (72%) responded to chemotherapy, and the 5-year overall survival was 71%, which indicates that the response to chemotherapy is likely to be a significant prognostic factor affecting overall survival after radical hepatic resection [15]. A prospective study of 50 patients with resectable CRLM who received neoadjuvant chemotherapy for 6 cycles found that the median recurrence-free survival was significantly influenced by tumor response (24.7 m for responding patients, 8.2 m for those with stable disease, and 3.0 m for those with progressive disease), which suggests that neoadjuvant chemotherapy may identify the best candidates for a potentially curative treatment approach [16]. Another Austrian study of 56 patients with potentially curable CRLM who received biweekly bevacizumab combined with chemotherapy for 6 cycles preoperatively demonstrated that objective response was increased by up to 73%, and 52 patients underwent liver resection without an increased rate of surgical or wound healing complications or severity of bleeding [17].

A recent systematic review of 23 studies comprising 3,278 patients identified an objective radiological response in 64% of patients after neoadjuvant chemotherapy, and a median of 9% and 36% had complete or partial pathological responses, respectively, whereas 41% had stable or progressive disease while receiving neoadjuvant chemotherapy; these results suggest that an objective response to neoadjuvant chemotherapy and improved disease-free survival (DFS) may be achieved in patients with resectable CRLM [18]. Therefore, based on a review of the best available evidence, an international panel of 21 experts in colorectal oncology comprised of liver surgeons and medical oncologists recommends that the majority of patients with CRLM should be treated up front with chemotherapy, irrespective of the initial resectability status of their metastases [19].

However, some studies do not support preoperative neoadjuvant chemotherapy in patients with resectable CRLM. A multicentric cohort study of 1471 patients resected for solitary, metachronous, primarily resectable CRLM without extrahepatic disease in the LiverMetSurvey International Registry conducted by Adam et al. demonstrated that the rate of postoperative complications was significantly higher in patients with neoadjuvant chemotherapy, and preoperative chemotherapy did not impact the overall survival or DFS [20]. Another study of 88 patients with neoadjuvant chemotherapy showed that there were no differences in the rates of concurrent extra-hepatic metastases observed intra-operatively but not on pre-operative imaging, whereas the size of intra-operatively observed CRLM was greater than that of CRLM identified from pre-operative imaging, and concluded that neoadjuvant chemotherapy for initially resectable CRLM does not reveal occult disease precluding surgical treatment [21].

In addition, numerous reports have reported that chemotherapy can be associated with significant changes to the hepatic parenchyma, which subsequently increase the risk of morbidity and mortality in the perioperative period [22]–[24]. Data from the current study, as well as data from prior studies, demonstrated that the incidence of morbidity in the NC group was higher than that in the WN group (33.9% vs. 25.8%), although there was no significant difference.

With the widespread use of highly efficient chemotherapy, some metastases may no longer be visible on imaging or during surgery, but a complete clinical response does not necessarily reflect a complete pathologic response. Thus, these patients should undergo hepatic surgery because long-term survival is expected. However, because they have no detectable lesions, they miss the best opportunity for liver surgery [25]. In the present study, none of the patients had disappearing liver metastases after neoadjuvant chemotherapy. Furthermore, in our clinic, patients are carefully monitored and receive surgery before metastases disappear.

To summarize, neoadjuvant chemotherapy administered before surgery to patients with initially resectable CRLM has the potential benefits and disadvantages [26], [27]. In the current study, our data suggest that not all patients with resectable CRLM will receive a survival benefit from preoperative neoadjuvant chemotherapy. After combination of literature review and data analysis, we found 4 independent prognostic factors for survival in these patients and divided them into a low-risk group and a high-risk group. In the high-risk group, the patients received a survival benefit from preoperative neoadjuvant chemotherapy. Thus, treatment of high-risk patients should start with chemotherapy. If the drugs are well chosen and the duration of treatment is carefully monitored during multidisciplinary meetings, the benefits largely outweigh the potential disadvantages.

This observation is important, since up to now researchers have continued to rely on clinical risk scores to compare outcomes following hepatectomy for colorectal liver metastases [28]. These clinical risk scores were derived in a different era when virtually no patients received effective preoperative chemotherapy and the definition of resectability was considered 1–3 unilobar metastases, resectable with a >1cm margin, and ideally detected metachronously. We now need validated new scoring systems that reflect contemporary practice both in terms of the definition of resectability and the use of highly effective chemo and biologic therapy regimens. And our study was a retrospective review with some limitations, such as selection bias and missing data. We are now validating our scoring system prospectively. However we recognize that we are still basing such scoring systems on morphologic characteristics that would have been familiar to Virchow (size, number, distribution etc.) more than a century ago, and we need to start to create scoring systems that reflect tumor biology.

## Conclusions

Preoperative neoadjuvant chemotherapy did not significantly increase mortality or complications. A primary tumor at stage T4, ≥4 liver metastases, the largest liver metastasis ≥5 cm in diameter, and a serum CEA level ≥5 ng/ml were independent prognostic factors for patients with resectable CRLM. Only resectable patients with more than 2 independent risk factors received a survival benefit from preoperative neoadjuvant chemotherapy.

## References

[1] Primrose JN (2010) Surgery for colorectal liver metastases. Br J Cancer 102: 1313–8. 20424612 10.1038/sj.bjc.6605659 PMC2865767

[2] Gilson N, Honoré C, Detry O, De Roover A, Coimbra C, et al (2009) Surgical management of hepatic metastases of colorectal origin. Acta Gastroenterol Belg 72: 321–6. 19902865

[3] Pinto Marques H, Barroso E, de Jong MC, Choti MA, Ribeiro V, et al (2012) Peri-operative chemotherapy for resectable colorectal liver metastasis: does timing of systemic therapy matter? J Surg Oncol 105: 511–9. 22065486 10.1002/jso.22133

[4] Choti MA, Sitzmann JV, Tiburi MF, Sumetchotimetha W, Rangsin R, et al (2002) Trends in long-term survival following liver resection for hepatic colorectal metastases. Ann Surg 235: 759–66. 12035031 10.1097/00000658-200206000-00002 PMC1422504

[5] Xu J, Qin X, Wang J, Zhang S, Zhong Y, et al (2011) Chinese guidelines for the diagnosis and comprehensive treatment of hepatic metastasis of colorectal cancer. J Cancer Res Clin Oncol 137: 1379–96. 21796415 10.1007/s00432-011-0999-8 PMC11828080

[6] Lupinacci R, Penna C, Nordlinger B (2007) Hepatectomy for resectable colorectal cancer metastases–indicators of prognosis, definition of resectability, techniques and outcomes. Surg Oncol Clin N Am 16: 493–506. 17606190 10.1016/j.soc.2007.04.014

[7] Kopetz S, Chang GJ, Overman MJ, Eng C, Sargent DJ, et al (2009) Improved survival in metastatic colorectal cancer is associated with adoption of hepatic resection and improved chemotherapy. J Clin Oncol 27: 3677–83. 19470929 10.1200/JCO.2008.20.5278 PMC2720081

[8] Adam R, Hoti E, Bredt LC (2010) Evolution of neoadjuvant therapy for extended hepatic metastases–have we reached our (non-resectable) limit? J Surg Oncol 102: 922–31. 21165994 10.1002/jso.21727

[9] Power DG, Kemeny NE (2010) Role of adjuvant therapy after resection of colorectal cancer liver metastases. J Clin Oncol 28: 2300–9. 20368552 10.1200/JCO.2009.26.9340

[10] Chua TC, Morris DL (2012) Resectable colorectal liver metastases: optimal sequencing of chemotherapy. J Gastrointest Cancer 43: 496–8. 21365476 10.1007/s12029-011-9272-2

[11] Nordlinger B, Sorbye H, Glimelius B, Poston GJ, Schlag PM, et al (2008) Perioperative chemotherapy with FOLFOX4 and surgery versus surgery alone for resectable liver metastases from colorectal cancer (EORTC Intergroup trial 40983): a randomised controlled trial. Lancet 371: 1007–16. 18358928 10.1016/S0140-6736(08)60455-9 PMC2277487

[12] Nordlinger B, Sorbye H, Glimelius B, Poston GJ, Schlag PM, et al (2012) EORTC liver metastases intergroup randomized Phase III Study 40983: long-term survival results. J Clin Oncol 30 (suppl) abstr 3508.

[13] Sorbye H, Mauer M, Gruenberger T, Glimelius B, Poston GJ, et al (2012) Predictive factors for the benefit of perioperative FOLFOX for resectable liver metastasis in colorectal cancer patients (EORTC Intergroup Trial 40983). Ann Surg 255: 534–9. 22314329 10.1097/SLA.0b013e3182456aa2

[14] Nasti G, Ottaiano A, Berretta M, Delrio P, Izzo F, et al (2010) Pre-operative chemotherapy for colorectal cancer liver metastases:an update of recent clinical trials. Cancer Chemother Pharmacol 66: 209–18. 20333385 10.1007/s00280-010-1297-x

[15] Chiappa A, Bertani E, Makuuchi M, Zbar AP, Contino G, et al (2009) Neoadjuvant chemotherapy followed by hepatectomy for primarily resectable colorectal cancer liver metastases. Hepatogastroenterology 56: 829–34. 19621711

[16] Gruenberger B, Scheithauer W, Punzengruber R, Zielinski C, Tamandl D, et al (2008) Importance of response to neoadjuvant chemotherapy in potentially curable colorectal cancer liver metastases. BMC Cancer 8: 120. 18439246 10.1186/1471-2407-8-120 PMC2386791

[17] Gruenberger B, Tamandl D, Schueller J, Scheithauer W, Zielinski C, et al (2008) Bevacizumab, capecitabine, and oxaliplatin as neoadjuvant therapy for patients with potentially curable metastatic colorectal cancer. J Clin Oncol 26: 1830–5. 18398148 10.1200/JCO.2007.13.7679

[18] Chua TC, Saxena A, Liauw W, Kokandi A, Morris DL (2010) Systematic review of randomized and nonrandomized trials of the clinical response and outcomes of neoadjuvant systemic chemotherapy for resectable colorectal liver metastases. Ann Surg Oncol 17: 492–501. 19856028 10.1245/s10434-009-0781-1

[19] Nordlinger B, Van Cutsem E, Gruenberger T, Glimelius B, Poston G, et al (2009) Combination of surgery and chemotherapy and the role of targeted agents in the treatment of patients with colorectal liver metastases: recommendations from an expert panel. Ann Oncol 20: 985–92. 19153115 10.1093/annonc/mdn735

[20] Adam R, Bhangui P, Poston G, Mirza D, Nuzzo G, et al (2010) Is perioperative chemotherapy useful for solitary, metachronous, colorectal liver metastases? Ann Surg 252: 774–87. 21037433 10.1097/SLA.0b013e3181fcf3e3

[21] Reddy SK, Tsung A, Marsh JW, Geller DA (2012) Does neoadjuvant chemotherapy reveal disease precluding surgical treatment of initially resectable colorectal cancer liver metastases? J Surg Oncol 105: 55–9. 21842519 10.1002/jso.22044

[22] Cleary JM, Tanabe KT, Lauwers GY, Zhu AX (2009) Hepatic toxicities associated with the use of preoperative systemic therapy in patients with metastatic colorectal adenocarcinoma to the liver. Oncologist 14: 1095–105. 19880627 10.1634/theoncologist.2009-0152

[23] Robinson S, Manas DM, Pedley I, Mann D, White SA (2011) Systemic chemotherapy and its implications for resection of colorectal liver metastasis. Surg Oncol 20: 57–72. 19962301 10.1016/j.suronc.2009.10.002

[24] Vauthey JN, Pawlik TM, Ribero D, Wu TT, Zorzi D, et al (2006) Chemotherapy regimen predicts steatohepatitis and an increase in 90-day mortality after surgery for hepatic colorectal metastases. J Clin Oncol 24: 2065–72. 16648507 10.1200/JCO.2005.05.3074

[25] Gaujoux S, Goéré D, Dumont F, Souadka A, Dromain C, et al (2011) Complete radiological response of colorectal liver metastases after chemotherapy: what can we expect? Dig Surg 28: 114–20. 21540596 10.1159/000323820

[26] Benoist S, Nordlinger B (2009) The role of preoperative chemotherapy in patients with resectable colorectal liver metastases. Ann Surg Oncol 16: 2385–90. 19554377 10.1245/s10434-009-0492-7

[27] Pawlik TM, Cosgrove D (2011) The role of peri-operative chemotherapy for resectable colorectal liver metastasis: what does the evidence support? J Gastrointest Surg 15: 410–5. 21253876 10.1007/s11605-011-1423-z PMC3547619

[28] Gomez D, Cameron IC (2010) Prognostic scores for colorectal liver metastasis: clinically important or an academic exercise? HPB (Oxford) 12: 227–38. 20590892 10.1111/j.1477-2574.2010.00158.x PMC2873645

